# A Pilot Metabolomic Study on Myocardial Injury Caused by Chronic Alcohol Consumption—Alcoholic Cardiomyopathy

**DOI:** 10.3390/molecules26082177

**Published:** 2021-04-09

**Authors:** Zhipeng Cao, Tianqi Wang, Wei Xia, Baoli Zhu, Meihui Tian, Rui Zhao, Dawei Guan

**Affiliations:** 1 Department of Forensic Pathology, School of Forensic Medicine, China Medical University, No. 77 Puhe Road, Shenyang North New Area, Shenyang 110122, China; zpcao@cmu.edu.cn (Z.C.); wangtianqidpq@outlook.com (T.W.); zhu1127@hotmail.com (B.Z.); tianmh0619@163.com (M.T.); 2 Department of Forensic Toxicological Analysis, School of Forensic Medicine, China Medical University, No. 77 Puhe Road, Shenyang North New Area, Shenyang 110122, China; lessenziale123@outlook.com

**Keywords:** alcoholic cardiomyopathy, metabolomics, differentially expressed metabolites, KEGG pathway, brain natriuretic peptide, myocardial injury, cardiac dysfunction

## Abstract

Chronic alcohol consumption leads to myocardial injury, ventricle dilation, and cardiac dysfunction, which is defined as alcoholic cardiomyopathy (ACM). To explore the induced myocardial injury and underlying mechanism of ACM, the Liber-DeCarli liquid diet was used to establish an animal model of ACM and histopathology, echocardiography, molecular biology, and metabolomics were employed. Hematoxylin-eosin and Masson’s trichrome staining revealed disordered myocardial structure and local fibrosis in the ACM group. Echocardiography revealed thinning wall and dilation of the left ventricle and decreased cardiac function in the ACM group, with increased serum levels of brain natriuretic peptide (BNP) and expression of myocardial BNP mRNA measured through enzyme-linked immunosorbent assay and real-time quantitative polymerase chain reaction (PCR), respectively. Through metabolomic analysis of myocardium specimens, 297 differentially expressed metabolites were identified which were involved in KEGG pathways related to the biosynthesis of unsaturated fatty acids, vitamin digestion and absorption, oxidative phosphorylation, pentose phosphate, and purine and pyrimidine metabolism. The present study demonstrated chronic alcohol consumption caused disordered cardiomyocyte structure, thinning and dilation of the left ventricle, and decreased cardiac function. Metabolomic analysis of myocardium specimens and KEGG enrichment analysis further demonstrated that several differentially expressed metabolites and pathways were involved in the ACM group, which suggests potential causes of myocardial injury due to chronic alcohol exposure and provides insight for further research elucidating the underlying mechanisms of ACM.

## 1. Introduction

With the development of the social economy, increased alcohol consumption has led to serious problems worldwide [1]. Alcoholic cardiomyopathy (ACM), the most common form of myocardial injury caused by chronic alcohol consumption, manifests as a progressive decrease in myocardial contractility and ventricular dilatation, leading to heart failure and arrhythmia [2]. ACM is the leading cause of nonischemic dilated cardiomyopathy in the United States and its prevalence in alcoholics is estimated to be approximately 21–32% [3]. Once heart failure occurs, the 4-year mortality rate is close to 50% if ACM patients continue to consume alcohol [4]. Although numerous studies on ACM have been conducted, the pathogenesis of ACM remains unclear.

Metabolomics is a new method of studying biological systems developed after genomics and proteomics. By analyzing small molecular substances in biological fluids and tissues, metabolomics reveals metabolic patterns and provides insight into the mechanisms underlying physiological conditions and diseases [5]. Considerable numbers of metabolomic studies have been conducted on cardiovascular diseases [6]. However, there are currently no metabolomic reports on myocardial injury due to chronic alcohol consumption. Therefore, the present study employed histopathology, echocardiography, molecular biology, and metabolomics to explore the myocardial injury induced by chronic alcohol consumption and the underlying mechanism of ACM.

## 2. Results

### 2.1. Morphological Changes in the Heart of ACM Model Mice

As shown in Figure S1 (Supplementary Materials), the heart/body weight ratio of mice in the ACM group (0.004260 ± 0.0001365) was statistically higher that in the control group (0.003729 ± 0.0001729) (*p* = 0.001).

**Figure 1:**
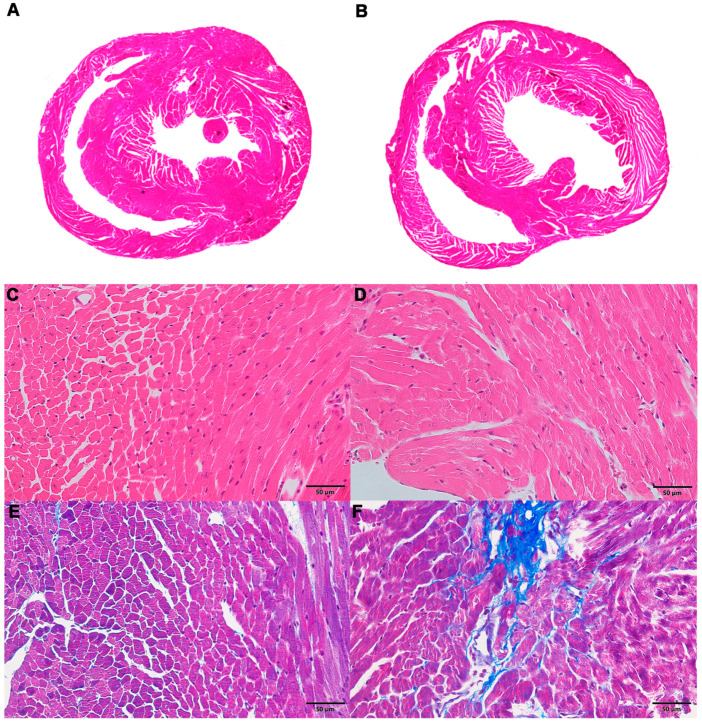
Morphological changes in the myocardium in the control and alcoholic cardiomyopathy (ACM) groups. (**A**) Representative hematoxylin-eosin (H&E) staining for the control group; (**B**) representative H&E staining for the ACM group; (**C**) representative H&E staining for the control group (400×); (**D**) representative H&E staining for the ACM group (400×); (**E**) representative Masson’s trichrome staining for the control group (400×); (**F**) representative Masson’s trichrome staining for the ACM group (400×).

Hematoxylin-eosin (H&E) staining of the myocardium revealed that the ventricles of mice in the ACM group were slightly dilated (Figure 1A,B), the cardiomyocytes showed slight hypertrophy, and the arrangement was locally disordered (Figure 1C,D) compared with the control group. Masson’s trichrome staining demonstrated a small number of collagen fibers around the vessels and myocardial interstitium in the myocardial tissue of mice in the ACM group, but these changes were not observed in the control group (Figure 1E,F). Sirius red staining revealed similar results with Masson’s trichrome staining (Figure S2).

### 2.2. Assessment of Cardiac Function

**Figure 2:**
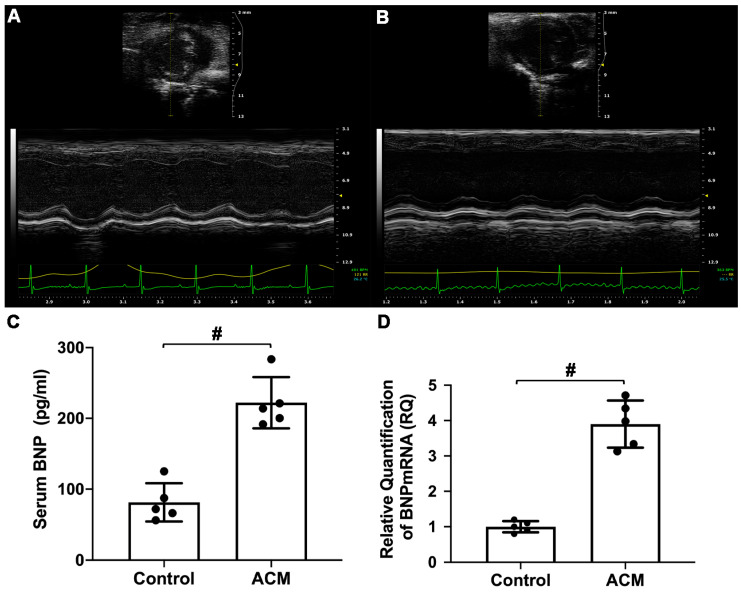
Assessment of cardiac function. (**A**) Echocardiography analysis of the control group; (**B**) echocardiography analysis of the ACM group; (**C**) biochemical analysis of serum brain natriuretic peptide (BNP) level; (**D**) analysis of myocardial BNP mRNA expression. # *p* < 0.01.

The results of echocardiographic analysis revealed that mice in the ACM group exhibited changes in cardiac dilatation and decreased cardiac function (Figure 2A,B). As shown in Table 1, the thicknesses of the anterior and posterior walls of the left ventricle at end-diastole and end-systole were significantly lower in the ACM group than in the control group. Additionally, the inner diameter and volume of the left ventricle at end-systole were significantly larger in the ACM group than in the control group. The cardiac ejection fraction (EF%) and fractional shortening (FS%) of the ACM group mice were significantly lower than those of the control group mice.

Serum brain natriuretic peptide (BNP) level and BNP mRNA expression in myocardial tissue were also used to assess cardiac function. Compared with the control group, serum BNP levels and myocardial BNP mRNA expression in the ACM group mice were significantly increased (Figure 2C,D).

### 2.3. Metabolic Pattern in the Myocardium of ACM Model Mice

#### 2.3.1. Sample Quality Control (QC)

The QC samples produced good Pearson’s correlation coefficients, indicating good stability of the test process and high data quality (Figure S3). The peaks extracted from all test and QC samples were processed by univariate scaling and then subjected to principal components analysis (PCA) analysis. The small difference between QC samples also indicated high data quality (Figure S4).

#### 2.3.2. PCA and Partial Least Squares Discriminant Analysis (PLS-DA) Results of the ACM and Control Groups

**Figure 3:**
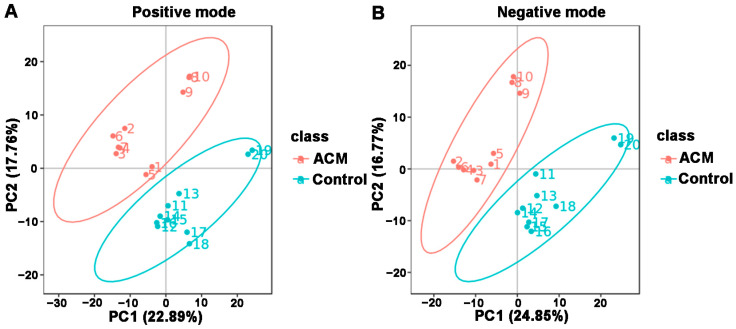
Principal components analysis (PCA) between the control and ACM groups. (**A**) Positive mode; (**B**) negative mode.

**Figure 4:**
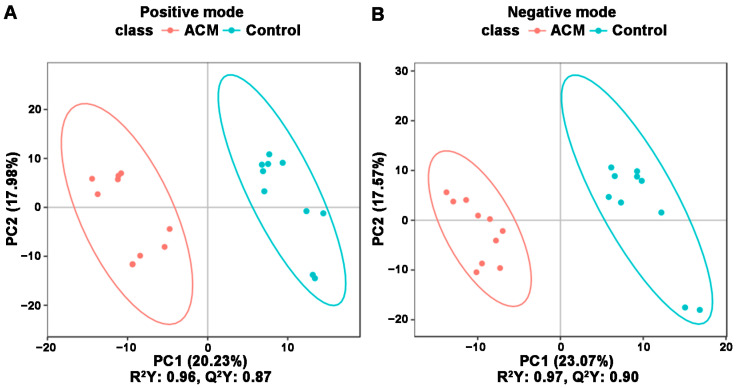
PLS-DA between the control and ACM groups. (**A**) Positive mode; (**B**) negative mode. Score plot from PLS-DA of the two compared groups. Each datapoint represents a function of the entire spectral profile of each subject.

The PCA and PLS-DA plots demonstrated satisfactory separation between the ACM and control groups (Figure 3 and Figure 4). Permutation tests of both positive and negative modes revealed that all Q^2^ values to the left were lower than the original points to the right, and the regression line of the Q^2^ points intersected the vertical axis below zero, which indicated no overfitting and good reliability of the PLS-DA models according to the validity criteria (Figure S5).

#### 2.3.3. Volcano Plot and Heatmaps of Differentially Expressed Metabolites

**Figure 5:**
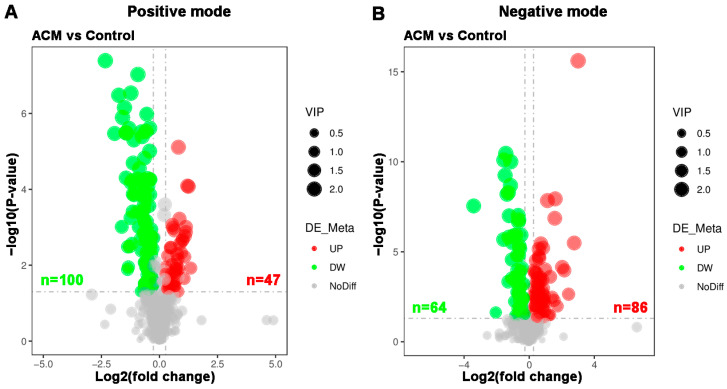
Volcano plots of myocardial metabolomics data between the control and ACM groups. (**A**) Positive mode; (**B**) negative mode. Red represents upregulated metabolites, green represents downregulated metabolites, and gray represents metabolites with no difference between the two groups. The variable importance in projection (VIP) values represent the most important metabolites obtained in the PLS-DA model.

**Figure 6:**
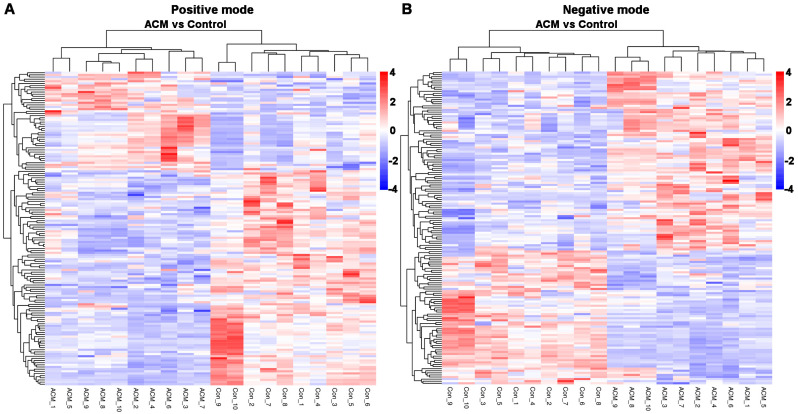
Heatmaps of differentially expressed metabolites between the control and ACM groups. (**A**) Positive mode; (**B**) negative mode.

A total of 297 differentially expressed metabolites were screened, of which 164 metabolites were downregulated and 133 metabolites were upregulated. The differentially expressed metabolites are displayed in a volcano plot (Figure 5), in which the gray dots represent indifferent metabolites, while the upregulated and downregulated metabolites are represented by red and green dots, respectively. Metabolic patterns are illustrated in the metabolite heatmaps (Figure 6).

#### 2.3.4. Enrichment of KEGG Pathway Analysis

**Figure 7:**
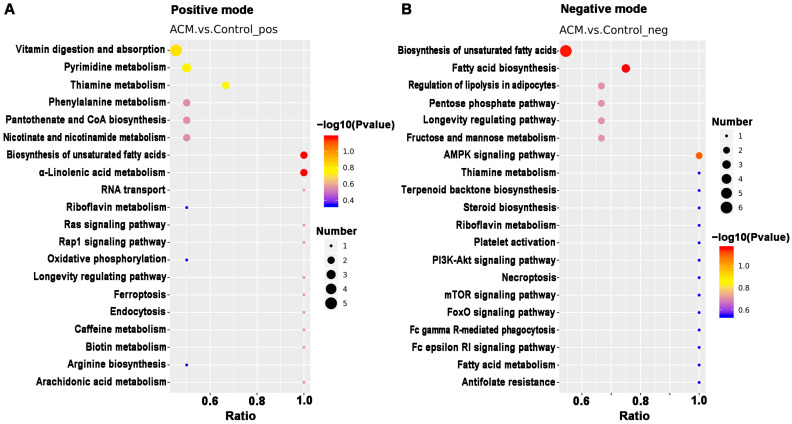
The top 20 KEGG pathways related to differentially expressed metabolites between the control and ACM groups. (**A**) Positive mode; (**B**) negative mode.

KEGG pathway analysis was applied to determine biochemical metabolic pathways and signal transduction pathways related to the differentially expressed metabolites between the ACM and control groups (Figure 7, Tables S1 and S2). The main KEGG pathways based on the differentially expressed metabolites are shown in Table 2. According to the enrichment results, ACM caused changes in the biosynthesis of the unsaturated fatty acids pathway, with downregulated levels of adrenic acid, docosapentaenoic acid (DPA), docosahexaenoic acid (DHA), and arachidonic acid and upregulated levels of alpha-linolenic acid, stearic acid, arachidic acid, and palmitic acid in the ACM group compared with the control group. Changes in the vitamin digestion and absorption pathways were also observed, corresponding to downregulated levels of thiamine monophosphate, pantothenic acid, nicotinamide, and riboflavin. The oxidative phosphorylation pathway, pentose phosphate pathway, and purine and pyrimidine metabolic pathways were also enriched. In addition, levels of some lipid metabolites were altered in the ACM group, including phosphatidylcholines (PCs), lysophosphatidylcholines (LysoPCs), and fatty acid esters of hydroxy fatty acids (FAHFAs).

## 3. Discussion

With improvements in socioeconomic status, the consumption of alcohol and incidence of ACM have gradually increased. In view of the morbidity and mortality of ACM, many studies have explored the pathogenesis of ACM in recent years. Although previous studies have reported metabolomics profiles after alcohol consumption, most studies have chosen serum or plasma as the research object [7]. However, systematic factors in a living organism may affect blood circulation, and the observed metabolic changes in circulating serum or plasma may not only be due to the myocardial injury induced by chronic alcohol consumption [8,9]. To the best of our knowledge, this is the first metabolomic study of myocardial injury due to chronic alcohol consumption.

**Figure 8:**
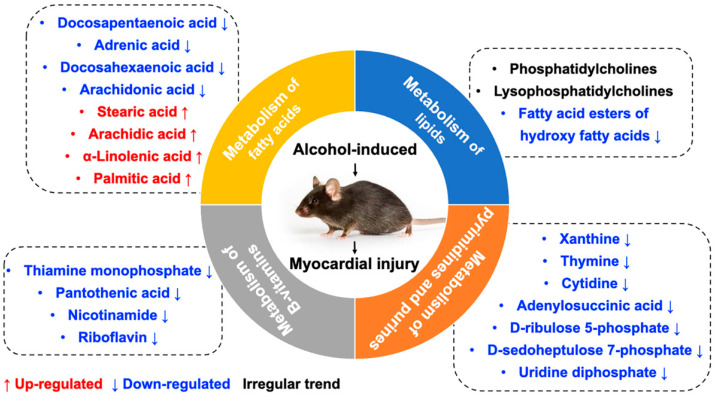
The main metabolites and their related KEGG pathways.

In the present study, morphological changes induced in myocardial tissues of ACM model mice were explored through H&E, Masson’s trichrome, and Sirius red staining, indicating disordered myocardial structure and local fibrosis. Echocardiography confirmed that the hearts of ACM mice were dilated with decreased cardiac function. Additionally, BNP, a widely used biomarker to diagnose heart failure in clinical practice, was also employed to assess cardiac function in ACM mice. Increased serum BNP levels and myocardial BNP mRNA expression in ACM mice verified decreased cardiac function. Individual differences in different batches of animals may lead to the discrepancies in some of the parameters of echocardiography, serum BNP levels, and BNP mRNA of myocardium compared to our previous report [1]. Despite all this, based on the results of echocardiography, histology, serum BNP levels, and BNP mRNA in myocardium, we can conclude that the ACM model was successfully established in the present study, which is the basis for further myocardial metabolomics study. Metabolomic investigation via ultra-high-performance liquid chromatography-tandem mass spectrometry (UHPLC-MS/MS) analysis screened out 297 differentially expressed metabolites which were involved in several key pathways through KEGG enrichment analysis. Herein, we discuss some of the major metabolites and their related KEGG pathways (Figure 8).

### 3.1. Metabolism of Fatty Acids

KEGG enrichment analysis revealed that many differentially expressed metabolites were associated with pathways related to fatty acid metabolism and biosynthesis of unsaturated fatty acids. Abnormal regulation of FAs has been frequently reported in cardiovascular diseases such as myocardial infarction and hypertrophy [6]. Studies of serum metabolomics have also demonstrated that free fatty acids can damage biological membranes and are partly responsible for the functional and morphological changes of alcohol-induced diseases [10,11].

Arachidonic acid reportedly inhibits the inflammatory response by binding to myeloid differentiation factor-2 (MD2) and preventing MD2/toll-like receptor 4 signaling activation to attenuate myocardial injury and fibrosis [12]. Similar to arachidonic acid, DHA can also inhibit saturated fatty acid-induced TLR4 activity and reactive oxygen species, thereby inhibiting inflammation [13]. Furthermore, DPA has been widely demonstrated to contribute to inhibition of platelet activation, anti-inflammatory effects, blood lipid improvement, and cardiovascular protective effects [14,15,16,17]. Conversely, prostaglandins, metabolites of arachidonic acid, have been widely proven to have proinflammatory effects [18]. Elevated levels of prostaglandin K1 (FC = 1.508, *p* = 0.005, VIP = 1.406) and K2 (FC = 6.798, *p* = 0.000, VIP = 2.021) were found in the present study, indicating proinflammatory effects on the myocardium caused by chronic alcohol consumption. In addition, palmitic acid, a saturated acid, can bind to TLR4 coreceptor MD2, induce inflammatory cytokines in cardiomyocytes, and is positively associated with incident heart failure [19,20,21]. Therefore, the downregulation of arachidonic acid, DPA, and DHA levels and the upregulation of palmitic acid and prostaglandins levels in myocardial tissue might induce inflammation in mice in the ACM group, which can further lead to fibrosis, myocardial remodeling, and cardiac dysfunction [22]. When heart function was affected, the mechanical stress on cardiomyocytes increased, which, in turn, led to increased BNP [23,24]. The disorder of free fatty acids might be one of the potential mechanisms of myocardial injury caused by chronic alcohol consumption.

### 3.2. Metabolism of Lipids

A metabolomics study on serum of rats who suffered from long-term alcohol exposure showed that alcohol affects mostly the lipid species in the serum of both female and male rats. The study also found that the glycerophospholipid, sphingolipid, and glycerolipids metabolism pathways; fatty-acyl profile of lipids; and total degree of unsaturation of fatty acid are profoundly altered by chronic alcohol exposure [25]. Abnormal lipid metabolism is closely related to the development of cardiovascular diseases [26,27,28]. The present study revealed many differentially expressed lipid metabolites, such as PCs and LysoPCs. However, many of them exhibited an irregular trend compared to previous studies which focused on circulating metabolites associated with alcohol intake in humans [29,30,31]. It is worth noting that levels of FAHFAs in the myocardial tissue of mice in the ACM group were significantly downregulated, although the relevant KEGG pathways were not enriched. FAHFAs, a new class of endogenous lipids derived from DHA, were first discovered in 2014 and have antidiabetic and anti-inflammatory effects in mammals [32,33,34]. Additionally, recent studies have determined that FAHFAs also have cardiovascular protective effects [35]. Although evidence for the direct interaction between alcohol and FAHFAs is deficient, downregulated FAHFA levels in the ACM group are speculated to be associated with the pathogenesis of myocardial injury induced by chronic alcohol consumption.

### 3.3. Metabolism of B Vitamins

Numerous studies have demonstrated that chronic alcohol consumption leads to B vitamin deficiency [36,37]. Compared with the control group, the myocardial tissue of mice in the ACM group exhibited decreased levels of several B vitamins, including thiamine (vitamin B1), riboflavin (vitamin B2), pantothenic acid (vitamin B5), and nicotinamide (the metabolite of niacin, vitamin B3). Among all B vitamins, thiamine deficiency is most associated with cardiovascular disease and cardiac dysfunction in patients with ACM [38]. Thiamine plays important roles in energy metabolism, antioxidation, and improvement of endothelial function, and thiamine deficiency can cause various diseases and dysfunctions, including heart failure [39,40]. Increased levels of metabolites related to oxidative phosphorylation were also observed in the ACM group, including nicotinamide adenine dinucleotide (NAD) and NAD+, which might be related to the downregulated levels of thiamine. Additionally, chronic alcohol consumption can also lead to riboflavin deficiency, which is also associated with heart failure [41,42]. Therefore, we speculate that extensive downregulation of B vitamins in the ACM group might be one of the potential mechanisms of ACM.

### 3.4. Metabolism of Pyrimidines and Purines

Compared with the control group, levels of d-ribulose 5-phosphate and d-sedoheptulose 7-phosphate of the pentose phosphate pathway were downregulated in the ACM group. One of the physiological significances of the pentose phosphate pathway is to supply important precursor substrates for purine and pyrimidine nucleotide synthesis [43]. Similarly, levels of metabolites involved in purine and pyrimidine metabolism, such as xanthine, thymine, cytidine, uridine diphosphate, and adenylosuccinic acid, were also decreased in the ACM group, indicating that alcohol affected nucleic acid synthesis of the cardiomyocytes. A recent study determined that levels of purine and pyrimidine metabolites were altered in patients with myocardial injury caused by anticancer drugs [44]. Further, levels of purine and pyrimidine metabolites reportedly changed in mice with acute myocardial ischemia, indicating that purine and pyrimidine metabolism disorders may be associated with myocardial injury [5]. Previous metabolomics studies have reported that alcohol exposure changed the level of hypoxanthine, but different studies have reported different trends [45,46,47]. Although the present study did not determine changes in hypoxanthine levels, a decrease in xanthine levels was observed. Combined with the above metabolites, we can determine that chronic alcohol consumption will change metabolism of pyrimidines and purines. However, the association between metabolism of pyrimidines and purines with myocardial hypertrophy and myocardial fibrosis in ACM individuals needs further study.

There were some limitations to the present study. First, the metabolites screened through metabolomics were not further verified. Second, only 10 myocardial specimens were used for metabolomics analysis, which only met the minimum requirements. In addition, studies have shown that alcoholism can affect the synthesis of fatty acids, thereby increasing the lipid infiltration of the cardiomyocytes [48,49]. However, in our study, no obvious myocardial lipid infiltration was observed by H&E staining. Unfortunately, due to the limitation of experimental conditions, we did not perform special staining for evaluating the fat infiltration of cardiomyocytes, such as oil-red O staining, which should be explored in future studies. Therefore, these results can be considered a pilot metabolomic study on myocardial injury after chronic alcohol exposure.

## 4. Materials and Methods

### 4.1. Chemical Reagents

Both liquid diets of 4% EtOH and non-EtOH Lieber–DeCarli were purchased from Trophic Animal Feed High-tech Co. Ltd. (Nanjing, China). Masson’s trichrome staining kits and H&E staining kit were from Beijing ZSGB Bio Co., Ltd. (Beijing, China). Sirius red staining solution was obtained from Beijing Solarbio Science & Technology Co., Ltd. (Beijing, China). The enzyme-linked immunosorbent assay (ELISA) kit of Mouse B-type natriuretic peptide (BNP) was from Cusabio Biotech Co., Ltd. (Wuhan, China). RNAiso Plus, SYBR^®^ Premix Ex Taq II, and the Kit of PrimeScriptTM RT Reagent were purchased from Takara Bio Inc. (Shiga, Japan). Methanol, liquid chromatography-mass spectrometry (LC-MS grade water), and formic acid were attained from prepared Thermo Fisher Scientific (Waltham, MA, USA).

### 4.2. Animal Model and Sample Collection

The Institutional Animal Care and Use Committee of China Medical University confirmed the animal assessments (IACUC Issue No. CMU2019266), which were consistent with the “Guide for the Care and Use of Laboratory Animals” (NIH Publication No. 86–23, Revised 1985).

Male mice (C57BL/6) with Specific Pathogen-Free (SPF)-grade (*n* = 30, 20–25 g, 7 weeks old) were attained from Beijing Vital River Laboratory Animal Technology Co., Ltd. (Beijing, China). These animals were randomized into control and ACM groups (*n* = 15 each) and accommodated in a facility with climate-controlled property within a dark/light 12 h cycle (3 mice per cage). The animal model used in this study was established as in previous studies [1]. Briefly, mice were acclimated to the alcoholic liquid diet via a gradient approach over a 1-week period based on provided instructions. Animals in the ACM group were administered a 4% EtOH (28% *w*/*v* of whole calories) Lieber–DeCarli liquid diet for a 12-week period, while control animals were administered a non-EtOH liquid diet with an equivalent calorie content over the same period.

Mice were killed after 12 weeks via cervical dislocation and blood specimens were accumulated through the puncture of cardiac. Serum was obtained by spinning blood for 15 min at 3000 rpm at 4 °C, and BNP biochemical analyses were conducted using these serum samples. In addition, samples of myocardial tissue were collected from 10 mice per group, rinsed with cold phosphate-buffered saline (PBS), and frozen at −80 °C for metabolomics analyses. The myocardial tissues from the remaining mice were used for histopathological staining and molecular experiments.

### 4.3. Histological Staining

Samples of myocardial tissue were fixed for 24 h with 4% paraformaldehyde, dehydrated via ethanol gradient, treated with xylene, and paraffin embedded. These tissues were then cut into the sections of about 5 μm, after which H&E, Masson’s trichrome, and Sirius red staining were carried out as per provided directions.

### 4.4. Echocardiography

A Vevo 2100 System with a mouse transducer of about 40 MHz (Visual Sonics, Toronto, ON, Canada) was used to conduct echocardiographic analyses as described previously [1]. Briefly, mice were anesthetized with a mixture of 100% O_2_ containing 1.5% isoflurane (flow rate 0.2 L/min) delivered via mask. The thickness, left ventricular fraction of ejection, volumes of end-diastolic and end-systolic, internal diameter of the left ventricle, and fraction of shortening of left ventricular were then analyzed. Mice were kept warm using a heating plate on a rail system.

### 4.5. ELISA

A BNP ELISA kit was used based upon provided directions to measure BNP levels in 50 uL of serum, with an ELX808 microplate reader (BioTek Instruments Inc., Winooski, VT, USA) being used to assess absorbance at 450 nm. 

### 4.6. Real-Time Quantitative PCR (qPCR)

After snap freezing in liquid nitrogen, 40 mg samples of myocardial tissues were ground into a powder, and RNA was separated from these samples with the RNAiso Plus reagent. The PrimeScript™ RT kit was then used to prepare cDNA using 10 μL containing 0.5 μL of PrimeScript RT Enzyme MixI, 0.5 μL of OligodT Primer (50 μM), 0.5 μL of Random 6 mers (100 μM), 2 μL of PrimeScript Buffer (5×), 4.5 μL of dH_2_O, and 2 μL of whole RNA (400 ng). A GeneAmp^®^ 9700 PCR system (Applied Biosystems, Foster City, CA, USA) was utilized for PCR amplification according to the thermocycler settings: 85 °C for 5 s, 37 °C for 15 min, and 4 °C for 5 min. All qPCR reactions were then conducted utilizing SYBR^®^ Premix Ex TaqTM II, as well as a system of 7500 Real-time PCR (Applied Biosystems, Foster City, CA, USA), with all mixtures of reaction comprising 10 μL of SYBR^®^Premix Ex TaqTM II (2×), 6 μL of dH_2_O, 0.4 μL of ROX Reference Dye II (50×), 0.8 μL of individual primers (10 μM), and 2 μL of cDNA. Thermocycler settings were as follows: 40 cycles at 95 °C for 5 s, 95 °C for 30 s, and 60 °C for 34 s. Primer sequences of BNP were: Forward 5′-AATTCAAGATGCAGAAGCTG-3′, reverse 5′-GAATTTTGAGGTCTCTGCTG-3′. Primer sequences of GAPDH were: Forward 5′-CTTTGTCAAGCTCATTTCCTGG-3′, reverse 5′-TCTTGCTCAGTGTCCTTGC-3′. The ΔΔCT was used to quantify relative BNP expression, with GAPDH serving as a normalization control.

### 4.7. Metabolite Extraction

Myocardial tissue specimens were pulverized in liquid nitrogen, after which they were mixed with 500 µL of 0.1% formic acid and cold 80% methanol and vortexed. Specimens were then spun down at 4 °C for 20 min at 15,000× *g*, and the dilution of supernatants was performed to an ultimate concentration of methanol of about 53% with LC-MS-grade water. Specimens were then spun down again at 4 °C for 20 min at 15,000× *g*, after which supernatants were utilized for LC-MS/MS analyses [50].

### 4.8. Metabolomic Analysis QC

Equivalent myocardial homogenate samples were combined together to yield QC samples, 3 of which were used to initially evaluate system performance. These QC samples were then regularly run between test samples to monitor variability and to facilitate QC data analysis. Following sample analysis, QC samples were analyzed in segments and the secondary spectra obtained for both test and QC samples were utilized for qualitative metabolite analyses, with test samples being analyzed in a random order to avoid batch effects [51]. 

The performance of the model of PLS-DA was evaluated by conducting 200 permutation tests, with overfitting of this model being assessed based upon R^2^ and Q^2^ values, which, respectively, corresponded to the goodness of fit and predictive power [52]. Validity was contingent upon total Q^2^ values to the left being lesser compared to the original on to the right, with the line of regression for these Q^2^ points intersecting the vertical axis below or at zero [53,54].

### 4.9. Analysis of UHPLC-MS/MS

UHPLC-MS/MS studies were conducted through Novogene Co., Ltd. (Beijing, China) with a system of Vanquish UHPLC (Thermo Fisher Scientific, Dreieich, Germany) and a mass spectrometer of Orbitrap Q ExactiveTM HF-X (Thermo Fisher Scientific, Waltham, MA, USA). Concisely, the injection of the specimens was executed onto a column of Hypersil Gold (Thermo Fisher Scientific, Waltham, MA, USA; 1.9 μm, 100 × 2.1 mm) with a linear gradient of 17 min at a flow rate of about 0.2 mL/min. Eluents A and B, namely 0.1% formic acid in aqueous milieu and methanol, respectively, were utilized. For the negative ion mode, eluents A and B, namely 5 mM ammonium acetate, pH 9.0, and methanol, respectively, were utilized. The settings of the elution of gradient were as follows: 2–100% B for 12.0 min, 2% B for 1.5 min, 100% B for 14.0 min, 100–2% B for 14.1 min, and 2% B for 17 min. The instrument of MS was employed in both positive and negative ion modes with a voltage of spray of around 3.2 kV, temperature of capillary of about 320 °C, flow rate of sheath gas of about 40 arb, and flow rate of auxiliary gas of about 10 arb.

### 4.10. Processing of Data and Identification of Metabolite

The Compound Discoverer software v3.1 (Thermo Fisher Scientific, Waltham, MA, USA) was utilized to analyze raw UHPLC-MS/MS data by conducting peak alignment, peak picking, and metabolite quantification by using the following criteria: time tolerance of retention = 0.2 min; actual tolerance of mass = 5 ppm; signal/noise ratio = 3; signal intensity tolerance = 30%; and minimum intensity = 104. Total intensity of spectrum was utilized to normalize peak intensities, and additive ion, fragment ion, and molecular ion peak formulas were predicted based upon normalized data. The mzCloud database (https://www.mzcloud.org/) (accessed on 31 December 2020), mzVault library, and mass lists were used for peak matching and obtaining accurate results. CentOS release 6.6, R v3.4.3, and Python v2.7.6 were applied to analyze the resultant data, with non-normally distributed data undergoing normal transformation where possible.

### 4.11. Statistical Analysis

The HMDB (https://hmdb.ca/metabolites) (accessed on 31 December 2020), KEGG (https://www.genome.jp/kegg/pathway.html) (accessed on 31 December 2020), as well as LIPID Maps (http://www.lipidmaps.org/) (accessed on 31 December 2020) databases were employed for metabolite annotation and analysis. PLS-DA and PCA were conducted with the MetaX computer program (http://metax.genomics.cn/) (accessed on 31 December 2020) Data were compared through Student’s *t*-tests, with metabolites considered to be differentially expressed if they exhibited an importance of variable for the projection (VIP) > 1 and a fold change (FC) ≥ 1.2, FC ≤ 0.83 or *p*-value < 0.05. Volcano plots of metabolite log_2_FC and −log10(*p*-value) values were generated to identify metabolites of interest. Z-scores of the areas of intensity related to the differentially expressed metabolites were used for normalization, and cluster heat maps were generated with the R pheatmap package. Pearson correlations between metabolites were assessed with the R cor() function, with significant correlations being identified with the cor.mtest() function. *p* < 0.05 was the significance threshold, and the corrplot R package was used to construct correlation plots. The KEGG database was used to assess the functions of differentially expressed metabolites and their related pathways, with pathway enrichment being defined as when x/*n* > y/*N*. Such enrichment was deemed significant at a *p* < 0.05 threshold.

Levels of serum BNP, BNP mRNA, and echocardiographic parameters were represented as means ± standard deviation (SD) and thoroughly compared via Student’s *t*-tests in SPSS 26.0 (IBM Corp., Armonk, NY, USA), with *p* < 0.05 as the significance threshold.

## 5. Conclusions

In conclusion, the present study demonstrated that chronic alcohol consumption caused disordered cardiomyocyte structure, thinning and dilation of the left ventricle, and decreased cardiac function. Metabolomic analysis of myocardium specimens and KEGG enrichment analysis further demonstrated that several differentially expressed metabolites in the ACM group were involved in pathways related to biosynthesis of unsaturated fatty acids, vitamin digestion and absorption, oxidative phosphorylation, pentose phosphate, and purine and pyrimidine metabolism. From the perspective of metabolomics, the present study provides insight into the causes of myocardial injury due to chronic alcohol exposure and lays a foundation for further research investigating its underlying mechanism.

## Figures and Tables

**Table 1:** The results of echocardiography.

Parameters	Control Group	ACM Group
LVAW; d (mm)	0.844 ± 0.044	0.595 ± 0.085 #
LVAW; s (mm)	0.877 ± 0.016	0.616 ± 0.042 #
LVPW; d (mm)	0.805 ± 0.085	0.601 ± 0.041 #
LVPW; s (mm)	0.999 ± 0.060	0.758 ± 0.044 *
LVID; d (mm)	4.165 ± 0.223	4.480 ± 0.226
LVID; s (mm)	3.164 ± 0.200	3.588 ± 0.142 *
LV Vol; d (uL)	77.290 ± 9.664	91.756 ± 11.157
LV Vol; s (uL)	40.032 ± 6.160	54.098 ± 5.234 *
EF%	48.244 ± 3.833	40.915 ± 1.713 *
FS%	24.049 ± 2.331	19.883 ± 1.041 *

# *p* < 0.01; * *p* < 0.05.

**Table 2:** The main KEGG pathways based on the differentially expressed metabolites.

KEGG Pathway	Screened Metabolite	FC	*p* Value	ROC	VIP	Trend	Mode
Biosynthesis of unsaturated fatty acids	Docosapentaenoic acid	0.451	0.000	1.000	2.054	down	negative
	Adrenic acid	0.465	0.000	1.000	1.936	down	positive
	Docosahexaenoic acid	0.514	0.000	1.000	1.910	down	negative
	Arachidonic acid	0.603	0.000	1.000	1.807	down	negative
	Stearic acid	1.225	0.000	0.970	1.646	up	negative
	Arachidic acid	1.454	0.001	0.900	1.461	up	negative
	α-Linolenic acid	1.493	0.001	0.880	1.587	up	positive
	Palmitic Acid	1.529	0.048	0.760	1.046	up	negative
Vitamin digestion and absorption	Thiamine monophosphate	0.519	0.000	1.000	1.883	down	positive
	Pantothenic acid	0.585	0.000	0.990	2.015	down	positive
	Nicotinamide	0.753	0.000	0.950	1.768	down	positive
	Riboflavin	0.788	0.001	0.920	1.588	down	positive
Metabolism of pyrimidines and purines	Xanthine	0.595	0.000	0.960	1.799	down	positive
	Thymine	0.708	0.032	0.940	1.194	down	positive
	Cytidine	0.756	0.000	0.990	1.819	down	positive
	Adenylosuccinic acid	0.820	0.017	0.790	1.238	down	negative
	d-Ribulose 5-phosphate	0.786	0.001	0.890	1.574	down	negative
	d-Sedoheptulose 7-phosphate	0.787	0.003	0.880	1.382	down	negative
	Uridine diphosphate	1.470	0.001	0.920	1.605	up	positive

## Data Availability

The data presented in this study are available in article and Supplementary Material.

## Supplementary Materials

The following are available online, Figure S1. The heart/body weight ratio (# *p* < 0.01). Figure S2. Representative Sirius red staining of myocardium (200×). (A) Control group (400×); (B) ACM group. Figure S3. Pearson’s correlations between quality control (QC) samples. (A) Positive mode; (B) Negative mode. Figure S4. Principal components analysis (PCA) among the control and ACM groups and QC samples. (A) Positive mode; (B) Negative mode. Figure S5. Permutation tests (200 times) performed in the PLS-DA model. (A) Positive mode; (B) Negative mode. PLS-DA, partial least squares discriminant analysis. Table S1. The main KEGG pathways based on the differentially expressed metabolites in positive mode. Table S2. The main KEGG pathways based on the differentially expressed metabolites in negative mode.

## Sample Availability

Samples are available from the authors.
