# Supplementary material for: A Pilot Metabolomic Study on Myocardial Injury Caused by Chronic Alcohol Consumption—Alcoholic Cardiomyopathy
Source: Molecules. 2021 Apr 9;26(8):2177. doi: 10.3390/molecules26082177 (PMC8070378; doi:10.3390/molecules26082177)
Supplement: Supplementary file 1 [file molecules-26-02177-s001.zip › molecules-1165985-supplementary/molecules-1165985-supplementary proofed/Supplementary Files/Supplementary Tables.pdf]

Table S1. The main KEGG pathways based on the differentially expressed metabolites in positive mode.

Name	FC	P value	ROC	VIP	Trend	KEGG pathway
Adrenic acid	0.465	0.000	1.000	1.936	down	Ferroptosis; Biosynthesis of unsaturated fatty acids
16(R)-HETE	0.468	0.000	0.960	1.781	down	Arachidonic acid metabolism
Thiamine monophosphate	0.519	0.000	1.000	1.883	down	Thiamine metabolism
Pantothenic acid	0.585	0.000	0.990	2.015	down	Vitamin digestion and absorption; Pantothenate and CoA biosynthesis
Xanthine	0.595	0.000	0.960	1.799	down	Caffeine metabolism; Purine metabolism
4-Oxoretinol	0.646	0.001	0.910	1.612	down	Retinol metabolism
Pantetheine	0.675	0.005	0.880	1.434	down	Pantothenate and CoA biosynthesis
Thymine	0.708	0.032	0.940	1.194	down	Pyrimidine metabolism
Phenylacetylglycine	0.727	0.032	0.800	1.144	down	Phenylalanine metabolism
Nicotinamide	0.753	0.000	0.950	1.768	down	Vitamin digestion and absorption; Nicotinate and nicotinamide metabolism
Cytidine	0.756	0.000	0.990	1.819	down	Pyrimidine metabolism
D-Phenylalanine	0.780	0.042	0.810	1.107	down	Phenylalanine metabolism
Riboflavin	0.788	0.001	0.920	1.588	down	Riboflavin metabolism; Vitamin digestion and absorption
GDP	0.821	0.004	0.880	1.434	down	RNA transport; Ras signaling pathway; Rap1 signaling pathway; Endocytosis
UDP	1.470	0.001	0.920	1.605	up	Pyrimidine metabolism
NAD+	1.472	0.032	0.740	1.119	up	Vitamin digestion and absorption; Longevity regulating pathway; Oxidative phosphorylation; Thiamine metabolism; Nicotinate and nicotinamide metabolism
α-Linolenic acid	1.493	0.001	0.880	1.587	up	Biosynthesis of unsaturated fatty acids; alpha-Linolenic acid metabolism
Traumatic acid	1.561	0.035	0.730	1.151	up	alpha-Linolenic acid metabolism

Table S2. The main KEGG pathways based on the differentially expressed metabolites in negative mode.

Name	FC	P value	ROC	VIP	Trend	KEGG pathway
Docosapentaenoic acid	0.451	0.000	1.000	2.054	down	Biosynthesis of unsaturated fatty acids
Docosahexaenoic acid	0.514	0.000	1.000	1.910	down	Biosynthesis of unsaturated fatty acids
Mevalonic acid	0.556	0.000	0.940	1.652	down	Terpenoid backbone biosynthesis; Ferroptosis
Arachidonic acid	0.603	0.000	1.000	1.807	down	Oxytocin signaling pathway; Necroptosis; Platelet activation; Fc epsilon RI signaling pathway; Fc gamma R-mediated phagocytosis; Retrograde endocannabinoid signaling; Long-term depression; GnRH signaling pathway; Linoleic acid metabolism; Vascular smooth muscle contraction; Inflammatory mediator regulation of TRP channels; Ferroptosis; Aldosterone synthesis and secretion; Biosynthesis of unsaturated fatty acids; Regulation of lipolysis in adipocytes
D-Mannitol 1-phosphate	0.673	0.000	0.960	1.848	down	Fructose and mannose metabolism
Galactinol	0.695	0.016	0.780	1.211	down	Galactose metabolism
Sedoheptulose 1,7-bisphosphate	0.728	0.004	0.870	1.415	down	Carbon metabolism
XMP	0.752	0.005	0.870	1.351	down	Purine metabolism
D-Ribulose 5-phosphate	0.786	0.001	0.890	1.574	down	Riboflavin metabolism; Pentose and glucuronate interconversions; Carbon metabolism; Pentose phosphate pathway
D-Sedoheptulose 7-phosphate	0.787	0.003	0.880	1.382	down	Pentose phosphate pathway; Carbon metabolism
Adenylosuccinic acid	0.820	0.017	0.790	1.238	down	Purine metabolism; Alanine, aspartate and glutamate metabolism
5-Aminovaleric acid	1.209	0.003	0.870	1.415	up	Lysine degradation
alpha-Ketoglutaric acid	1.211	0.025	0.780	1.147	up	Alanine, aspartate and glutamate metabolism; Butanoate metabolism; Glyoxylate and dicarboxylate metabolism; Pentose and glucuronate interconversions; D-Glutamine and D-glutamate metabolism
Stearic acid	1.225	0.000	0.970	1.646	up	Biosynthesis of unsaturated fatty acids; Fatty acid biosynthesis
Dulcitol	1.286	0.004	0.860	1.368	up	Galactose metabolism
Decanoic acid	1.430	0.030	0.720	1.088	up	Fatty acid biosynthesis
Arachidic acid	1.454	0.001	0.900	1.461	up	Biosynthesis of unsaturated fatty acids

Nicotinamide adenine dinucleotide	1.465	0.020	0.850	1.217	up	Thiamine metabolism; AMPK signaling pathway; Longevity regulating pathway; Aldosterone synthesis and secretion; Oxidative phosphorylation
Cyclic ADP-ribose	1.484	0.011	0.890	1.287	up	Salivary secretion; Pancreatic secretion; Oxytocin signaling pathway
Calcitriol	1.520	0.016	0.780	1.164	up	Steroid biosynthesis; Endocrine and other factor-regulated calcium reabsorption; Mineral absorption; Tuberculosis
Adenosine 5'-monophosphate	1.523	0.006	0.810	1.356	up	AMPK signaling pathway; Longevity regulating pathway; Aldosterone synthesis and secretion; Antifolate resistance; FoxO signaling pathway; mTOR signaling pathway; PI3K-Akt signaling pathway; Olfactory transduction; Taste transduction; cGMP-PKG signaling pathway; Renin secretion; Purine metabolism; Regulation of lipolysis in adipocytes
Palmitic acid	1.529	0.048	0.760	1.046	up	Biosynthesis of unsaturated fatty acids; Fatty acid biosynthesis; Fatty acid metabolism; Fatty acid elongation
Succinic acid	1.909	0.003	0.860	1.377	up	Propanoate metabolism; Sulfur metabolism; Oxidative phosphorylation; Alanine, aspartate and glutamate metabolism; Butanoate metabolism; Glyoxylate and dicarboxylate metabolism
Xanthurenic acid	3.031	0.000	1.000	2.001	up	Tryptophan metabolism
L-Fucose	8.029	0.000	1.000	2.161	up	Fructose and mannose metabolism
