# Supplementary figures and images for: A Pilot Metabolomic Study on Myocardial Injury Caused by Chronic Alcohol Consumption—Alcoholic Cardiomyopathy
Source: Molecules. 2021 Apr 9;26(8):2177. doi: 10.3390/molecules26082177 (PMC8070378; doi:10.3390/molecules26082177)

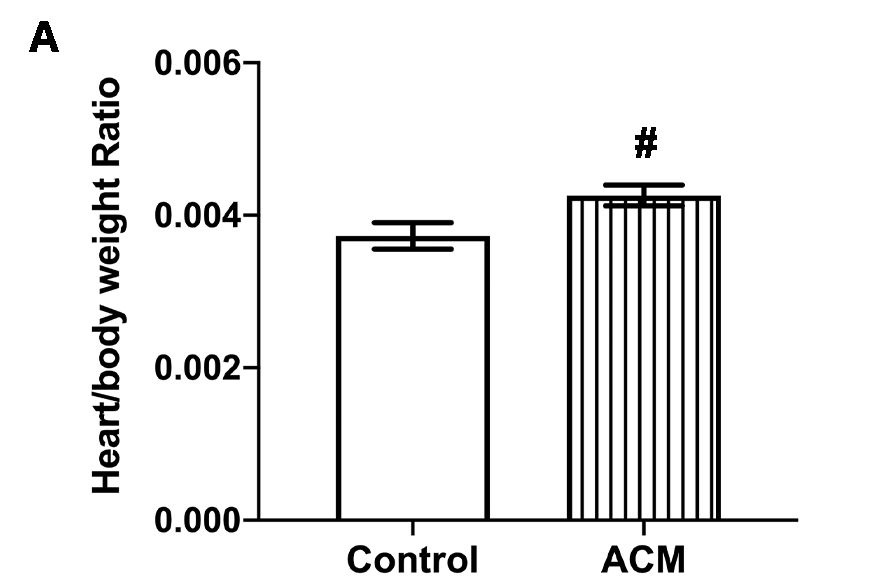

Supplement: Supplementary file 1 [file molecules-26-02177-s001.zip › molecules-1165985-supplementary/molecules-1165985-supplementary proofed/Supplementary Files/Figure S1.tiff]

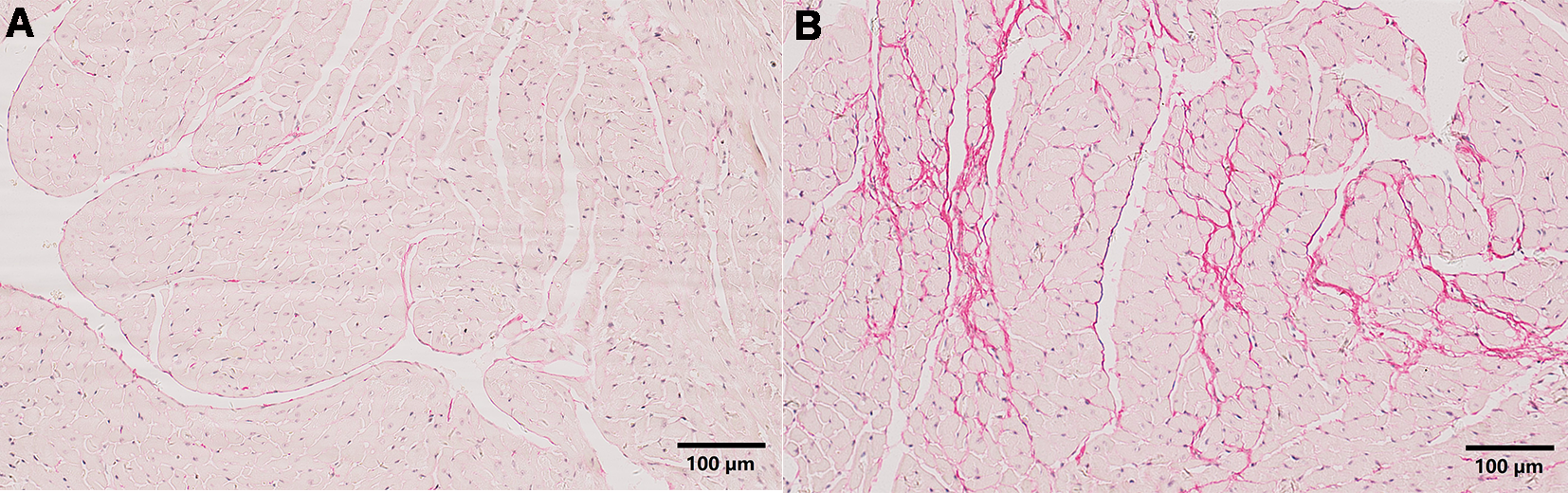

Supplement: Supplementary file 1 [file molecules-26-02177-s001.zip › molecules-1165985-supplementary/molecules-1165985-supplementary proofed/Supplementary Files/Figure S2.tiff]

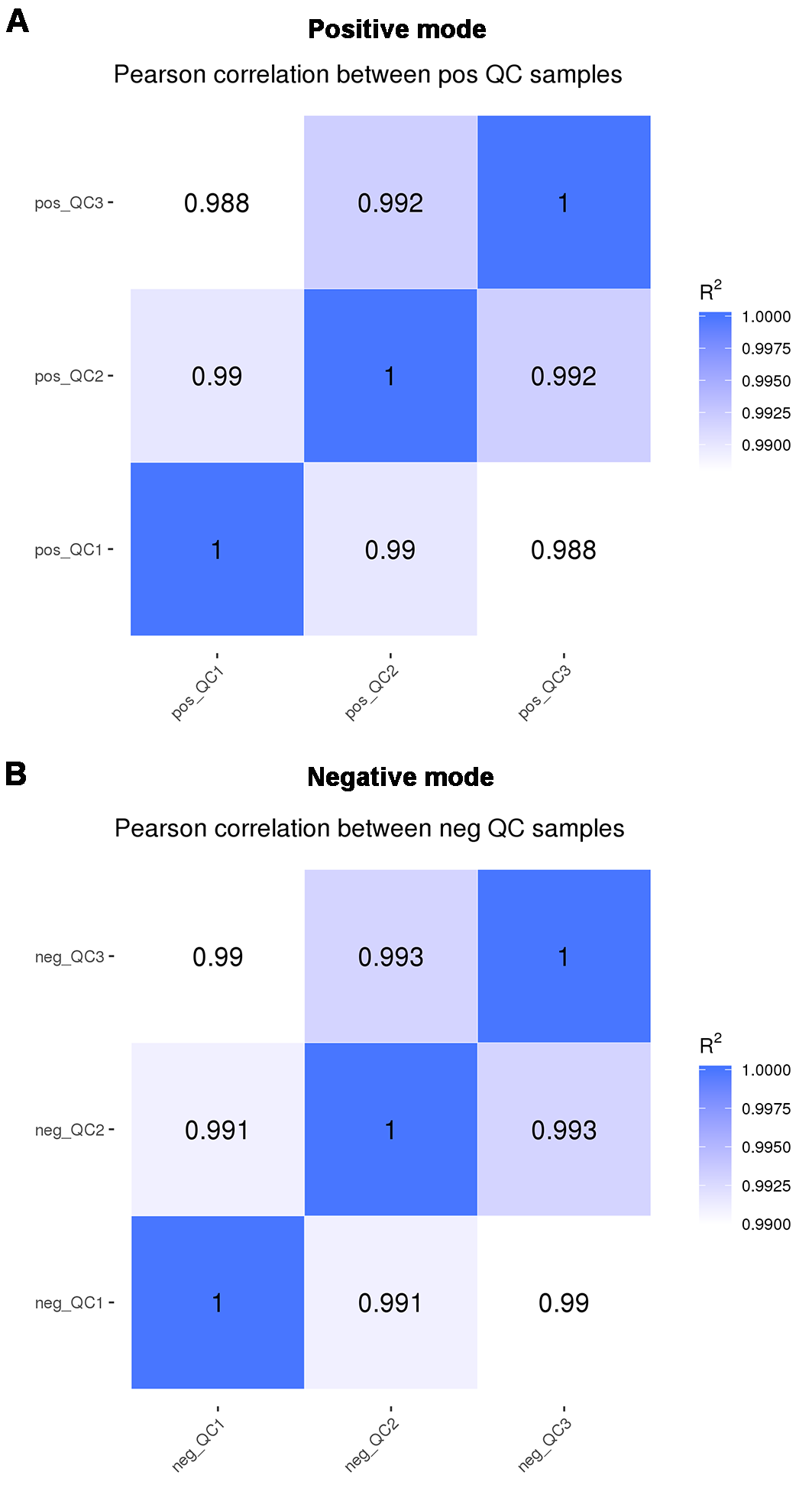

Supplement: Supplementary file 1 [file molecules-26-02177-s001.zip › molecules-1165985-supplementary/molecules-1165985-supplementary proofed/Supplementary Files/Figure S3.tiff]

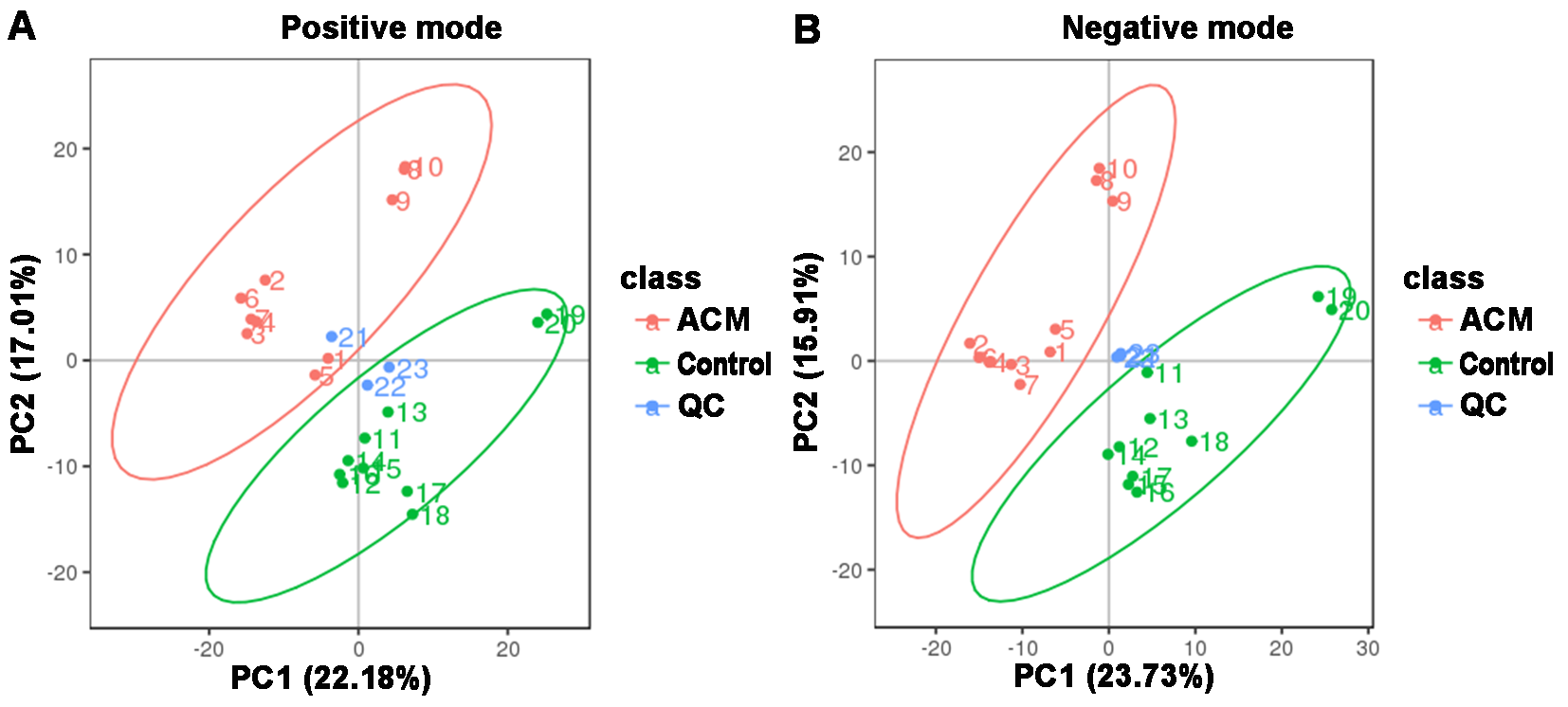

Supplement: Supplementary file 1 [file molecules-26-02177-s001.zip › molecules-1165985-supplementary/molecules-1165985-supplementary proofed/Supplementary Files/Figure S4.tiff]

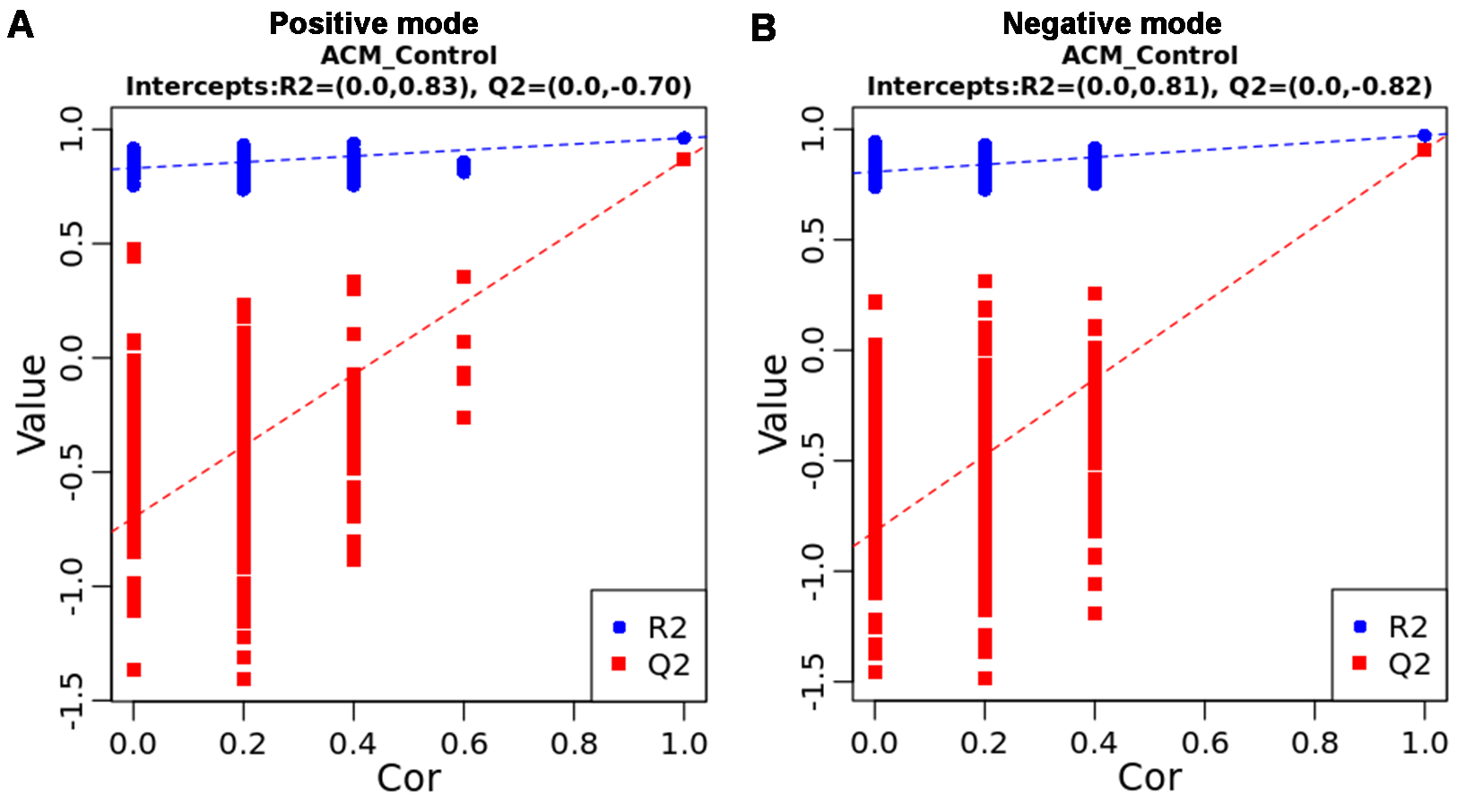

Supplement: Supplementary file 1 [file molecules-26-02177-s001.zip › molecules-1165985-supplementary/molecules-1165985-supplementary proofed/Supplementary Files/Figure S5.tiff]
